# Implications of gestational age at antenatal care attendance on the successful implementation of a maternal respiratory syncytial virus (RSV) vaccine program in coastal Kenya

**DOI:** 10.1186/s12889-020-09841-9

**Published:** 2020-11-16

**Authors:** Joyce U. Nyiro, Elizabeth Bukusi, Dufton Mwaengo, David Walumbe, Amek Nyaguara, Bryan Nyawanda, Nancy Otieno, James A. Berkley, Patrick Munywoki, D. James Nokes

**Affiliations:** 1 Kenya Medical Research Institute (KEMRI)-Wellcome Trust Research Programme, Centre for Geographic Medicine Research-Coast, P.O Box 230-80108, Kilifi, Kenya; 2 Kenya Medical Research Institute (KEMRI), Centre for Microbiology Research, Nairobi, Kenya; 3 University of Nairobi, Institute of Tropical and Infectious Diseases, Nairobi, Kenya; 4 Kenya Medical Research Institute (KEMRI), Centre for Global Health Research, Kisumu, Kenya; 5 School of Life Sciences and Zeeman Institute (SBIDER), University of Warwick, Coventry, UK

**Keywords:** Pregnant women, Antenatal care attendance, Gestational age, Maternal respiratory syncytial virus vaccine, Effectiveness

## Abstract

**Background:**

Maternal immunisation to boost respiratory syncytial virus (RSV) specific antibodies in pregnant women is a strategy to enhance infant protection. The timing of maternal vaccination during pregnancy may be critical for its effectiveness. However, Kenya has no documented published data on gestational age distribution of pregnant women attending antenatal care (ANC), or the proportion of women attending ANC during the proposed window period for vaccination, to inform appropriate timing for delivery or estimate potential uptake of this vaccine.

**Methods:**

A cross-sectional survey was conducted within the Kilifi Health and Demographic Surveillance System (KHDSS), coastal Kenya. A simple random sample of 1000 women who had registered pregnant in 2017 to 2018 and with a birth outcome by the time of data collection was taken. The selected women were followed at their homes, and individually written informed consent was obtained. Records of their antenatal attendance during pregnancy were abstracted from their ANC booklet. The proportion of all pregnant women from KHDSS (55%) who attended for one or more ANC in 2018 was used to estimate vaccine coverage.

**Results:**

Of the 1000 women selected, 935 were traced with 607/935 (64.9%) available for interview, among whom 470/607 (77.4%) had antenatal care booklets. The median maternal age during pregnancy was 28.6 years. The median (interquartile range) gestational age in weeks at the first to fifth ANC attendance was 26 (21–28), 29 (26–32), 32 (28–34), 34 (32–36) and 36 (34–38), respectively. The proportion of women attending for ANC during a gestational age window for vaccination of 28–32 weeks (recommended), 26–33 weeks and 24–36 weeks was 76.6% (360/470), 84.5% (397/470) and 96.2% (452/470), respectively. Estimated vaccine coverage was 42.1, 46.5 and 52.9% within the narrow, wide and wider gestational age windows, respectively.

**Conclusions:**

In a random sample of pregnant women from Kilifi HDSS, Coastal Kenya with card-confirmed ANC clinic attendance, 76.6% would be reached for maternal RSV vaccination within the gestational age window of 28–32 weeks. Widening the vaccination window (26–33 weeks) or (24–36 weeks) would not dramatically increase vaccine coverage and would require consideration of antibody kinetics data that could affect vaccine efficacy.

**Supplementary Information:**

The online version contains supplementary material available at 10.1186/s12889-020-09841-9.

## Background

Respiratory syncytial virus (RSV) is the main cause of severe lower respiratory tract infections among infants 0–5 months of age [1, 2]. Globally, RSV is estimated to have caused 1·4 million hospital admissions for acute lower respiratory infections and 27,300 in-hospital deaths of infants under 6 months of age in 2015, with 96% of these occurring in developing countries [3]. Analysis from a multi-site etiology study conducted in 2011–2014 in sub-Saharan Africa and Asia, revealed that nearly 40% of all hospital admissions with severe or very severe pneumonia among infants under 1 year, were caused by RSV [1].

With the highest burden of RSV disease in early infancy, particularly in those under 3 months of life [4], a vaccine to administer in the first few weeks of life would appear to be the most logical target for RSV disease prevention. However, development of such a vaccine has faced major difficulties such as poor immunological responses and reactogenicity to vaccines in this age group [5–7]. Thus to date there are no licenced childhood vaccines for RSV. A preventive strategy which involves providing prophylaxis to infants at birth and during RSV season is in advanced stages of clinical trials (NCT03959488). Trials which intramuscularly administered a long acting RSV Prefusion F-targeting monoclonal antibody (MEDI8897) in healthy preterm infants showed the monoclonal antibody to be safe and protective against medically attended RSV [8, 9].

Maternal vaccination is currently considered the most plausible strategy for the near term to protect these infants [10–12]. Several candidate maternal RSV vaccines are advancing in phase 2 and phase 3 clinical trials [13, 14]. A RSV F subunit protein vaccine design is progressing towards late stage trials (NCT04032093). A maternal RSV vaccine candidate of nanoparticle design (NCT02624947) completed phase 3 of clinical trials in early 2019. According to results of the phase 3 trial, the vaccine prevented RSV associated disease hospitalization in young infants 3 months of age, by 44.4% (95% CI; 19.6–61.5%). The trial results also showed that mothers immunized < 33 weeks of gestational age had higher vaccine efficacy across all endpoints [15, 16]. In a study for maternal Tetanus-Diphtheria-acellular pertussis vaccine, anti-pertussis IgG avidity was found to be higher when the vaccine was administered during 28–32 weeks gestation [17].

Following progress in development of maternal RSV vaccines, it is considered that these vaccines will be particularly beneficial in low income countries due to the disproportionate RSV disease burden. A gap analysis report (https://www.path.org/resources/roadmap-advancing-rsv-maternal-immunization/) on advancing maternal immunization, proposes introduction of maternal RSV vaccines in low- and middle-income countries (LMICs) through the ANC platform. However, maternal characteristics including ANC attendance patterns among women in LMICs are different from those in high income countries where efficacy trials are conducted. The level of RSV specific antibodies rapidly wanes over time [18] and decline to pre-infection levels within 3 months [19]. Therefore, timing of a maternal RSV vaccine delivery should be within a window of gestational age that will result in maximum benefit to the infant. Accurate gestational age information from pregnant women attending for ANC screening in LMICs is required to inform the timing of vaccine delivery and expected vaccine coverage. Data on multiple ANC visits is lacking in sub-Saharan Africa countries, since the National Demographic Health Surveys (DHS) focus on collecting data from the first ANC visit only [20].

In this study, we aim to describe the distribution of gestational age at each attendance for ANC care among pregnant women from the population of the Kilifi Health and Demographic Surveillance System (KHDSS) area, Coastal Kenya. We also describe the proportions attending ANC during a proposed vaccination window, we estimate the maternal RSV vaccine coverage and how this may influence the successful implementation of a maternal RSV vaccine program in this setting.

## Methods

### Study population

The study was conducted in Kilifi County, coastal Kenya at the KEMRI Wellcome Trust Research Programme (KWTRP). Collection of gestational age data was carried out within the KHDSS area. The KHDSS, described elsewhere in detail [21, 22], was established in 2000 by KWTRP for the purposes of demographic surveillance and epidemiological research. The area under surveillance comprises, administratively, 15 locations and 40 sub-locations, covering an area of 890km^2^, extending 35 km north and south of Kilifi County Hospital (KCH) in Kilifi town [22]. Currently, the system monitors a population of around 300,000 residents through enumeration rounds conducted three times a year. The number of pregnancies occurring per year is registered during these enumeration rounds. The crude birth rate is approximately 8000 live births per year [22]. A survey asking all pregnant women in the KHDSS, if they attended ANC was introduced into the pregnancy monitoring questionnaire in 2018.

KHDSS census registers were used to select a random sample of women to participate in this study. A standard method [23] was used to determine the sample size by which to estimate the proportion of women attending each ANC visit with a precision of +/− 5%. A sample size of 384 women was determined using an assumption that the gestational age of each of the recommended four ANC individual visits by pregnant women from Kilifi HDSS population would be representative of the general population of pregnant women in coastal Kenya. This relates to the median gestational age at first ANC visit obtained from the 2014 Kenya Demographic Health Survey of 24 weeks [20].

To overcome limitations of missing data due to missed ANC visits and unavailable ANC booklets from KHDSS women residents, initially estimated at 60% (unpublished data from KHDSS pregnancy monitoring survey), a total of 1000 women were randomly selected from the census register within the KWTRP integrated database. The study included women with a pregnancy registered in 2017 and 2018 census rounds and who had a birth outcome by the time of data collection (October 2018 to February 2019). These women were traced and visited at their homes by trained fieldworkers. Informed consent was sought and if a woman was willing to participate, she was requested to present her ANC booklet. Records on gestational age at attendance for ANC care, tetanus vaccine uptake, birth outcomes and other demographic details were extracted from the ANC booklets. Gestational age in the ANC booklets was estimated by fundal height. Additional information on socio-cultural factors and other obstetric history was collected using a standardized electronic questionnaire (Additional file 1) loaded in computer tablets. For women not found at home during the first visit, two more follow-ups were made and further attempt to locate them through other household members were conducted, after which they were confirmed to be not available for interview.

## Ethical considerations

Informed consent was obtained in writing from all participants. This study was approved by the KEMRI Scientific and Ethical Review Unit Committee (SERU #3716).

## Statistical analyses

Data were analysed in STATA version 13.1 (College Station, Texas). Gestational age dating in weeks was measured by fundal height which is standard practice in all public health facilities in Kenya. Trimester was defined as 1 (1–12 weeks), trimester 2 (13–26 weeks) and trimester 3 (27–42 weeks). A further three gestational age categories were generated by which to assess ANC initiation, i.e. < 16 weeks, early initiation as recommended by Kenya national guidelines for quality obstetric care [24], 16–28 weeks: mid initiation (timing of second visit), 29–42 week: late initiation which corresponds with timing of third and fourth ANC visits. Gestational ages in weeks at ANC visits were presented as mean (Standard Deviation; (SD)), median and interquartile range (IQR). Proportions of women attending ANC during three potential gestational age windows for vaccine delivery (i.e. 28–32 weeks, 26–33 weeks and 24–36 weeks) were calculated. For each of the three defined vaccination windows, namely gestational age of pregnancy ranging from 28 to 32 weeks or 26 to 33 weeks or 24 to 36, the number of women attending ANC was computed and presented as a proportion of all women with ANC attendance records. Vaccine coverage was estimated as a product of the proportion of women attending ANC during a potential gestational age window for maternal RSV vaccine delivery and the proportion of all women from the KHDSS with birth outcomes registered in 2018 and who reported to have attended ANC. A chi-square test was performed to assess the association between gestational age at first ANC attendance and maternal characteristics. The characteristics of women within the KHDSS area with and without ANC booklets were compared using the chi-square test. Density curves for the distribution of gestational age at ANC attendance were generated.

## Results

**Fig. 1:**
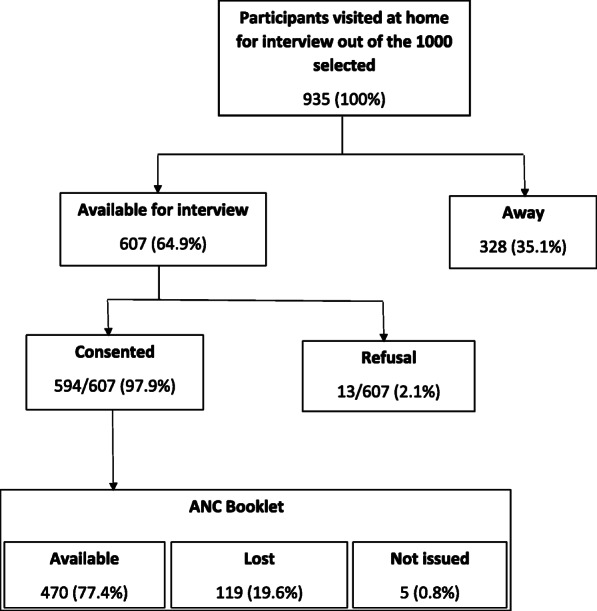
Flow chart showing sampling of women from Kilifi Health and Demographic Surveillance System area who participated in the study

Of the 1000 women selected from the KHDSS database, 935 were visited at their home of whom 607/935 (64.9%) were available for interview. Of those available, 594/607 (97.9%) consented to enroll into the study. Overall, 470/607 (77.4%) reported their ANC booklet was available, 119/607 (19.6%) reported it was lost and 5/607 (0.8%) said it was not issued (Fig. 1). The median (interquartile, IQR) age at the time of pregnancy was 28.6 years (23.4–33.6). The youngest was 14.5 years while the oldest was 48.3 years. About a third of these women, 228 (38.4%), were either in their first or second pregnancy. The highest parity was 13 pregnancies.

### Characteristics of pregnant women with and without ANC booklets

There were no significant differences between pregnant women with (*n* = 470) and without (*n* = 124) ANC booklets in the following demographic characteristics: maternal age (χ^2^, *P* = 0.431), education level (χ^2,^ *P* = 0.238), occupation (χ^2^, *P* = 0.266), marital status (χ^2^, *P* = 0.288), religion (χ^2^, *P* = 0.081) and number of pregnancies (χ^2^, *P* = 0.189). Significant difference was identified for place of delivery (χ^2^, *P* = 0.015). Women without ANC booklets were more likely to have delivered at home (38.7% vs 27.5%) (Table 1).

**Table 1:** Characteristics of women with and without ANC booklets sampled from the Kilifi Health Demographic Surveillance System (KHDSS) area, Coastal Kenya

Characteristics	With booklet n (%)	Without booklet n (%)	Total N (%)	Chi2 ***P*** value
**N**	470 (79.1)	124 (20.9)	594 (100)	
**Age class**				
15–19	24 (5.1)	5 (4.0)	29 (4.9)	
20–24	115 (24.5)	29 (23.4)	144 (24.2)	
25–29	110 (23.4)	21 (16.9)	131 (22.1)	0.431
30–34	116 (24.7)	35 (28.2)	151 (25.4)	
35–39	66 (14.0)	16 (12.9)	82 (13.8)	
40–44	34 (7.23)	15 (12.1)	49 (8.25)	
45–49	6 (1.3)	2 (1.6)	8 (1.4)	
**Marital status**				
Married	434 (92.3)	111 (89.5)	545 (91.8)	
Single	34 (7.2)	11 (8.9)	45 (7.5)	0.288
Divorced/Sep/Widowed	2 (0.4)	2 (1.6)	4 (0.7)	
**Delivery place**				
Health facility	341 (72.6)	76 (61.3)	417 (70.2)	0.015
Home	129 (27.5)	48 (38.7)	177 (29.8)	
**Education level**				
**None**	82 (17.5)	17 (13.7)	99 (16.7)	
Primary	326 (69.4)	97 (78.2)	423(71.2)	0.238
Secondary	48 (10.2)	7 (5.7)	55(9.3)	
Tertiary-College/University	14 (3.0)	3 (2.4)	17 (2.9)	
**Gravida**				
1–2	188 (39.8)	40 (32.3)	228 (38.4)	
3–5	159 (33.8)	43 (33.8)	201 (33.8)	0.189
6–9	110 (23.4)	39 (31.5)	149 (25.1)	
10–15	14 (3.0)	2 (1.6)	16 (2.7)	

### Distribution of gestational age at ANC visits among pregnant women in Kilifi

**Fig. 2:**
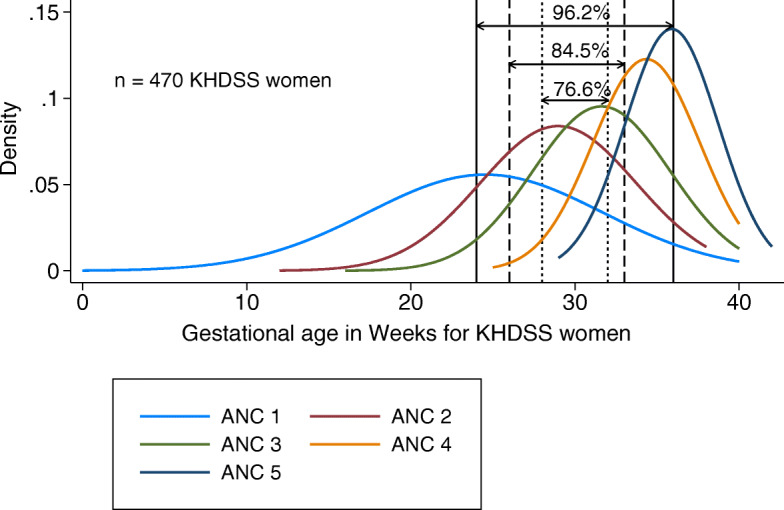
Density distribution curves of gestational age by ANC visit among women sampled from the KHDSS area. Each curve represents participant’s ANC visits i.e. visit one to fifth. The three gestational age windows (28–32 weeks), (26–33 weeks) and (24–36 weeks) for maternal RSV vaccination and the proportion of women attending within that gestational age window (76.6, 84.5 and 96.2% respectively), are also shown

**Fig. 3:**
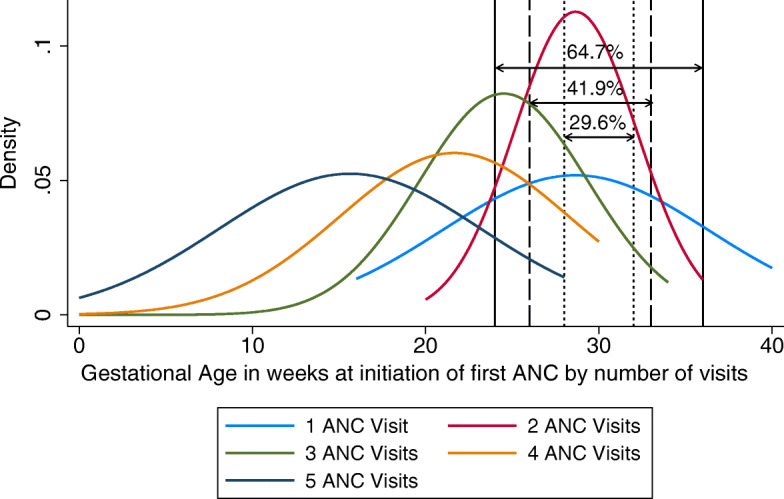
Density distribution curves of gestational age in weeks at initiation of first ANC visit by number of ANC visits attended among women sampled from the KHDSS area. Each curve represents participant’s number of ANC visits i.e. one visit to five visits. Three gestational age windows (28–32 weeks), (26–33 weeks) and (24–36 weeks) for maternal RSV vaccination and the proportion of women attending within that gestational age window during the first ANC visit (29.6, 41.9 and 64.7% respectively), are also shown

The distribution of how pregnant women in Kilifi attended for ANC screening is shown using density curves in Fig. 2 and Fig. 3. Gestational age at first ANC visit varied widely and progressively diminished with increasing ANC visit number (Fig. 3). Some women attended for first ANC care at less than 10 weeks of gestation while others visited in their 40th week of pregnancy.

**Fig. 4:**
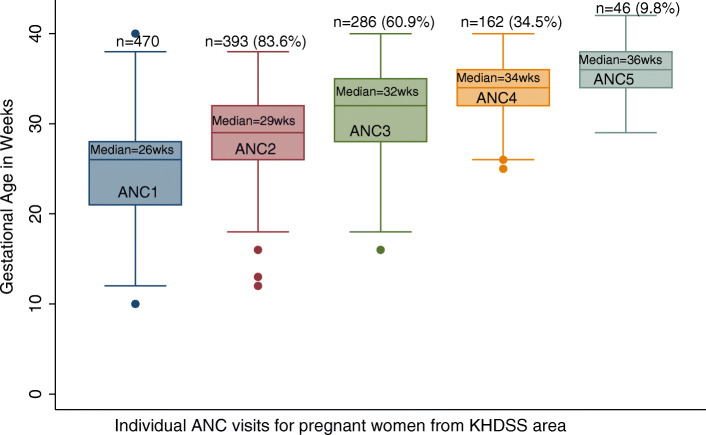
A box plot showing the gestational age in weeks against ANC visits among participants from the KHDSS area. Each box represents ANC visit from first to fifth (i.e. ANC1, ANC2, ANC3, ANC4 and ANC5). The median and proportion of women out of the 470 participants with ANC booklets attending ANC in each visit is also shown

The median gestational age among women at attendance for first to fifth ANC visit was 26 weeks (IQR: 21–28), 29 weeks (26–32), 32 weeks (28–35), 34 weeks (32–36) and 36 weeks (34–38), respectively (Fig. 4).

The mean (SD) gestational age in which women attended for their first to fifth ANC care was 24.5 (7.2) weeks, 28.9 (5.0) weeks, 31.6 (4.2) weeks, 34.3 (3.2) weeks and 35.9 (2.8) weeks, respectively (Table 2).

**Table 2:** Summary of gestational age in weeks by ANC visit and proportion available for vaccination within a specific window among pregnant women interviewed from Kilifi Health Demographic Surveillance System area, Coastal Kenya

ANC Visit	Participant’s n (%)	Mean Gest age (weeks)	95% Confidence Interval (CI)	Median Gest age (weeks)	IQR Gest age (weeks)	Proportion VAC-Window1 (28–32 weeks	Proportion VAC-Window2 (26–33 weeks)	Proportion VAC-Window3 (24–36 weeks)
ANC1	470 (100)	24.5	23.9–25.2	26	21–28	29.6	41.9	64.7
ANC2	393 (83.6)	28.9	28.4–29.4	29	26–32	39.8	48.9	72.3
ANC3	286 (60.8)	31.6	31.1–32.1	32	28–35	26.2	31.9	52.9
ANC4	162 (34.5)	34.3	33.8–34.8	34	32–36	10	12.3	26.4
ANC5	46 (9.8)	36.9	35.0–36.7	36	34–38	1.1	1.3	6.6
**Total**	**470**					**76.6**	**84.5**	**96.2**

The proportion of women attending for first to fifth ANC visit is shown in Fig. 4. A total of 83.6% (393/470) attended second ANC, 60.9% (286/470) third ANC, 34.5% (162/470) fourth ANC and 9.8% (46/470) attended a fifth ANC visit. (Fig. 4).

The median gestational age at first ANC visit among women who attended one, two, three, four or five visits was 30 weeks (IQR: 26–34), 28 weeks (26–31), 24 weeks (22–28), 23 weeks (20–26) and 18 weeks (12–20), respectively (Table 3).

**Table 3:** Summary of gestational age in weeks at initiation of first ANC visit by number of visits attended and proportion available for vaccination within a specific window during the first visit among pregnant women interviewed from Kilifi Health Demographic Surveillance System area, Coastal Kenya

Number of ANC Visits	Participants n (%)	Median Gest age (weeks) at First ANC visit	IQR Gest age (weeks) at first ANC visit	Proportion VAC-Window1 (28–32 weeks) at first ANC visit	Proportion VAC-Window2 (26–33 weeks) at first ANC visit	Proportion VAC-Window3 (24–36 weeks) at first ANC visit
1	77 (16.4)	30	26–34	6.0	7.5	12.8
2	107 (22.8)	28	26–31	13.6	17.2	21.5
3	124 (26.4)	24	22–28	6.6	10.2	18.1
4	116 (24.7)	23	20–26	3.2	6.8	11.7
5	46 (9.8)	18	12–20	0.2	0.2	0.6
**Total**	**470 (100)**			**29.6**	**41.9**	**64.7**

The proportion of women who attended one, two, three, four or five ANC visits was 16.4% (77/470), 22.8% (107/470), 26.4%(124/470), 24.7%(116/470) and 9.8%(46/470), respectively.

The proportion of all pregnant women from the KHDSS with birth outcomes registered in 2018 who reported to have attended at least one ANC was 55%. This proportion was obtained from pregnancy monitoring data collected during enumeration rounds.

### Proportion of women attending ANC within a specific gestational age window for maternal RSV vaccine delivery

The proportion of pregnant women participants with ANC booklets, who attended for ANC during the narrow (28–32 weeks gestation period) and wide (26–33 weeks) or wider (24–36 weeks gestation period), was 76.6% (360/470), 84.5% (397/470) and 96.2% (452/470), respectively, Table 2. Of the women attending an ANC clinic during weeks 28–32 gestational age, 29.6% were attending for their first visit, 39.8% for their second, 26.2%, for their third, 10% for their 4th and 1% for their 5th. For the 26–33 weeks gestational age window, close to half (48.9%) were attending for their second visit. For the wider vaccination window of 24–36 weeks gestation, 64.7% were in their first ANC visit, 72.3% in their second, 52.9% third and 6.6% in their fifth visit (Table 2). The proportion of pregnant women who attended only one ANC visit and would have been reached for vaccination at the gestational age window of 28–32 weeks, 26–33 weeks and 24–36 weeks was 6.0%(28/470),7.5%(35/470) and 12.8%(60/470) respectively (Table 3).

### Tetanus vaccine coverage during the proposed maternal RSV vaccination window

Pregnant women received either a single or multiple doses of tetanus vaccine as they attended for ANC care. Out of the 470 women with ANC booklets, 18 (3.8%) received a tetanus in their first trimester, 206 (43.8%) in the second trimester and 298 (63.4%) during the third trimester (27–42 weeks gestation). A total of 257 (54.7%) women received tetanus vaccine within the potential gestational age window period for maternal RSV vaccination of 28–32 weeks and 284 (60.4%) during the wide window of 26–33 weeks. Overall, 316/470 (67.2%) of the women received a tetanus vaccine (either one or more doses) during ANC visits.

### Factors influencing ANC initiation and uptake of health services among pregnant women in Kilifi

The proportion of women attending for first ANC visit while in their first, second or third trimester of pregnancy were 4.9% (23/470), 53.8% (253/470) and 41.3% (194/470) respectively. A very low proportion of the women (5.9%; 28/470) had an early initiation of first ANC visit of less than 16 weeks as recommended by the Kenya national guidelines for quality obstetric care [24]. Delay on initiation of first ANC visit was significantly associated with being of older maternal age (i.e. 35 years and above) at the time of pregnancy (χ^2^, *P* = 0.022), education below secondary level (χ^2^, *P* = 0.021) and preference for home births (χ^2^, *P* < 0.001). We did not find any significant association between gestational age at first ANC visit and marital status (χ^2^, *P* = 0.798), travel distance (in kilometres) from home of the participant to the ANC health facility (χ^2^, *P* = 0.436), gravida (χ^2^, *P* = 0.078) and religion (χ^2^, *P* = 0.477) (Table 4).

**Table 4:** Characteristics of participants by gestational age at first ANC visit in Kilifi Health Demographic Surveillance System area, Coastal Kenya

Participants Gestational age at First ANC visit	0–15 weeks	16–28 weeks	29–42 weeks	Total n (%)	Chi2 P value
**Total participants (%)**	28 (5.9)	319 (67.9)	128 (26.2)	470 (100)	
**Age class**					
15–19	1 (4.2)	19(79.2)	4 (16.7)	24 (5.1)	
20–24	5 (4.4)	83(72.2)	27 (23.5)	115 (24.5)	
25–29	9 (8.2)	81 (73.6)	20 (18.2)	110 (23.4)	0.022
30–34	4 (3.5)	72 (62.6)	39 (33.9)	115 (24.5)	
35–39	6 (9.1)	42 (63.6)	18 (27.3)	66 (11.0)	
40–44	1 (2.9)	20 (58.8)	13 (38.2)	34 (7.23)	
45–49	2 (33.3)	2 (33.3)	2 (33.3)	6 (1.3)	
**Gravida**					
1–2	12 (6.4)	142 (75.5)	34 (18.1)	188 (39.8)	0.078
3–5	9 (5.7)	102 (64.6)	47 (29.8)	158 (33.8)	
6–9	6 (5.5)	67 (60.9)	37 (33.6)	110 (23.4)	
10–15	1 (7.1)	8 (57.2)	5 (35.7)	14 (3.0)	
**Distance to ANC health facility (Kms)**					
0–5	15 (5.2)	198 (68.8)	75 (26.0)	288 (66.5)	
6–10	3 (4.0)	52 (69.3)	20 (26.7)	75 (17.8)	0.436
11–20	3 (7.1)	33 (78.6)	6 (14.3)	42 (9.8)	
21–30	0 (0)	6 (54.6)	5 (45.4)	11 (2.6)	
31–40	1 (11.1)	4 (44.4)	4 (44.4)	9 (2.1)	
40–70	1 (16.7)	4 (66.7)	1 (16.7)	6 (1.4)	
**Education level**					
**None**	3 (3.7)	51 (62.2)	28 (34.1)	82 (17.5)	
Primary	19 (5.8)	220 (67.5)	87 (26.7)	326 (69.4)	0.021
Secondary	3 (6.2)	37 (77.1)	8 (16.7)	48 (10.2)	
Tertiary-College/University	3 (21.4)	11 (78.6)	0 (0)	14 (2.9)	
**Delivery place**					
Health facility	25 (7.3)	248 (72.7)	68 (19.9)	341 (72.6)	0.001
Home	3 (2.33)	71 (55.0)	55 (42.6)	129 (27.4)	
**Marital status**					
Married	26 (6.0)	296 (68.2)	112 (25.8)	434 (92.4)	
Single	2 (5.9)	21 (61.8)	11 (32.3)	34 (7.2)	0.798
Divorced/Sep/Widowed	0 (0)	2 (100.0)	0 (0)	2 (0.4)	

When the 594 women were asked about the decision on timing for ANC screening when pregnant, 564 (94.9%) reported having made their own decision on when to attend for ANC screening. However, 30 (5%) reported they consulted either spouse or relative. The great majority of women interviewed (585, 98.5%) reported they attended ANC for the wellbeing of themselves and their unborn child. Six (1.0%) reported it was because of pregnancy complications while the remainder were following advice from friends and relatives.

Among those who delivered at home (177 women, 29.8%), a total of 58 (32.8%) of the participants reported that this was due to the distance to the health facility, 47 (26.6%) reported it was as a result of doctors’ and nurses’ strikes, while 71 (40.1%) had other reasons. There was a strong association between education level and choice of place for delivery (χ^2^, *P* < 0.001). A majority of women with primary level of education (8 years of formal education) or less had their babies delivered at home.

## Discussion

Maternal immunisation to boost RSV specific antibodies, is a strategy proposed to protect infants against RSV associated disease within the first few months of life [15, 25]. Implementation of a maternal RSV vaccine program will be influenced by several factors, one of them being the appropriate timing of vaccination. The window of gestational age at vaccination that will result in optimal maternal RSV-specific antibody transfer to the infant depends on antibody kinetics. However, a major unknown is the distribution of gestational age at ANC visits that determines the proportion of women who attend at the ideal window for vaccination. Here we provide a detailed analysis of the gestational age at attendance for ANC screening, the proportion attending ANC within a specific gestational age window for vaccine delivery and describe the related factors among pregnant women in Kilifi.

A random sample of women was selected from the registers of a demographic surveillance area. Not all women had an ANC booklet. Those with or those without ANC booklet did not differ in most characteristics (e.g. maternal age, occupation, education level, marital status, religion and number of pregnancies), but did differ in place of delivery. This was an important observation to address potential bias in the results that would have been associated with sampling of the participants or missing data.

We found initiation of first ANC visit (median 26 weeks) among KHDSS women is later than the WHO guidelines for a first visit of first 12 weeks’ gestation [26] and as recommended by the Kenya national guidelines for quality obstetric care [24]. Although we found the delay in ANC initiation to be associated with other multiple factors, late presentation for first ANC screening limited the number of visits a pregnant woman could attend. Kenya still implements the basic ANC model of four ANC visits [24] which recommend first visit less than 16 weeks of gestation, second visit between 16 to 28 weeks, third visit between 28 to 32 weeks, and fourth visit between 32 to 40 weeks [24]. The WHO recommends pregnant women to have a total of 8 contacts with a health care provider [26]. In this study, about 10% of the women with ANC booklets attended five ANC visits. Nevertheless, even at the 5th ANC visit, at least 1% were still within the proposed gestational age window for vaccination. Whereas, among the 77 women who attended only one ANC visit in this study, 40 of them would have been missed for maternal RSV vaccination. This emphasizes the need to encourage pregnant women to attend multiple ANC visits in order to increase the opportunity of receiving all required health services, including vaccination.

We computed estimates of the proportion of pregnant women that would be reached for vaccination if delivery is through ANC clinics. We estimated 76.6% of pregnant women (with at least one ANC visit) from the KHDSS area were within the gestational age window period targeted for vaccination of 28–32 weeks. Widening the vaccination window to 26–33 weeks and 24–36 weeks could see the proportion increase to 84.5 and 96.2%, respectively. The current maternal RSV vaccines in clinical trials are antibody boosting vaccines [13, 14]. Previous studies have shown that, maternal RSV antibodies from the KHDSS population decay rapidly at the rate of − 0.58 (SD: 0.20) log^2^PRNT titre per month [18] and reach very low levels within a period of three months [19]. The gestational age window for maternal RSV vaccination is therefore defined to occur within a time when delivery of the vaccine will ensure there is efficient maternal antibody transfer, such that the level rising from boosting and the antibody decay kinetics combine to provide the infant maximum benefit. A study to assess effect of timing of Tetanus-Diphtheria-acellular pertussis vaccine administration in pregnancy showed that vaccination during 28–32 weeks gestation was associated with higher anti-pertussis IgG avidity, as compared with vaccination during 33–36 weeks gestation [17]. Incorporation of antibody kinetics data to the wide and wider potential gestational age windows for maternal RSV vaccine delivery will therefore be necessary to confirm vaccine efficacy.

We estimated the maternal RSV vaccine coverage in this population using pregnancy data for ANC attendance collected during enumeration rounds from all KHDSS pregnant women. This is because we did not have estimates of pregnant women who reported not to have attended ANC from the selected sample. A pilot survey within the KHDSS, asking all women with birth outcomes if they attended ANC, showed that 55% of the women who had birth outcomes in 2018 attended for at least one ANC visit. The KHDSS area records about 8000 pregnancies per year [22]. Assuming 55% of the 8000 KHDSS pregnant women attended ANC and 76.6% are available for vaccination during the gestational age window of 28–32 weeks, the vaccine coverage among all pregnant women within KHDSS area would be 42.1%. A gestational age window of 26–33 weeks, with 84.5% visiting in the vaccine window, would increase the overall vaccine coverage to 46.5%. A window of gestational age of between 24 and 26 weeks with 96.2% of women attending ANC, will have 52.9% vaccine coverage.

In this study, we used maternal tetanus vaccine to assess the proportion of pregnant women that will be reached for vaccination if there is concomitant administration with the maternal RSV vaccine through ANC platform. We approximated 54.7% of the women in Kilifi attending ANC who received a tetanus vaccine would be reached for maternal RSV vaccination within the gestational age window period of 28–32 weeks. This implies, the estimated coverage for maternal RSV vaccine will be 30.1% if it is co-administered with tetanus vaccine in this setting. However, we find these estimates lower than the maternal tetanus vaccine coverage reported by the ministry of health, District Health Information System (DHIS2) for Kilifi County of 45% for the year 2018. We note here that, co-administration of multiple vaccines during ANC attendance is possible though there might be challenges in obtaining accurate estimates of the vaccine coverage. This is likely if one of the vaccines is not influenced by the gestational age at the time of delivery and is limited in the number of recommended lifetime doses one should be eligible for, such as the tetanus vaccine.

We also found that, factors that influence utilization of health care services can also influence the level of success of a new intervention delivered through health facilities. In this study, we found women who had home births reported the main reasons as long distance to a health facility and health-care workers strikes. Notably, most of these women did not have education beyond primary school level. A study in Ghana reported socio-cultural perceived threats to pregnancy forced women to seek care during pregnancy from multiple sources including traditional herbalists [27]. While, a study among Ethiopian women showed that education status at primary level was associated with home deliveries [28]. In our analysis, we did not find travel distance to the health facility to be related to timing for initiation of ANC visit (*P* = 0.436) or choice of a place for delivery. We also do not think the cost of accessing maternal care might directly hinder utilization of health services among Kilifi women. This is because there are initiatives in Kenya (“Beyond Zero”) [29] to provide free access to maternal and child health services for all pregnant women, but 30% of births in this study were reported to have occurred at home. The fact that some women declined to disclose reasons for not seeking obstetric care during pregnancy, shows there could be other underlying and unknown factors that could impact negatively the delivery of any new maternal vaccine program. Multiple initiatives as a strategy to encourage and influence positive health care seeking practices might be useful for this population. We recommend inclusion of reproductive health education early in school and further studies that can engage women who prefer having home deliveries to understand the key issues restraining them from utilizing health care services.

There are some limitations to this study. The first and major limitation is that, pregnancy dating by fundal height is not an accurate method for estimating gestational age. Fundal height is likely to under or overestimate gestational age in this setting. However, this is the method available and in use in all public hospitals in Kenya. Pregnancy dating by last menstrual period which is dependent on participant recall was missing for most women. Second, only 47% of the random sample of 1000 pregnant women had ANC booklets available. Therefore, our sample might not be representative of the general KHDSS pregnant women population. We tried to assess for this bias using demographic data from our census registers and we found these women were similar in most characteristics. Third, women without available ANC booklets or those who deliver at home may still have visited ANC clinics and could therefore potentially receive a vaccine. Fourth, the estimated vaccination window can only infer vaccine coverage and not necessarily vaccine effectiveness. Whether within this proposed window, the infant will have maximum benefit requires incorporation of data on antibody kinetics. In addition, vaccine effectiveness will require assessment of data on birth weight, gestational age at delivery, prematurity, as well as health factors like maternal malaria, anaemia, HIV infection, hypergammaglubinemia etc., which this study could not address. The present study provides insights into the distribution of gestational age and proportion of pregnant women likely to be reached for vaccination. Further work is currently ongoing which will incorporate this data and data on antibody kinetics from women in this population. This will be used in mathematical modelling to estimate the optimal gestational age for maternal RSV vaccine delivery. To our knowledge, this is the first study from Kenya and sub-Saharan Africa to present data on timing for ANC visits which includes gestational age for subsequent visits and describes how this timing in ANC attendance is likely to affect the success of a maternal RSV vaccine program. These data can also be relevant in other maternal vaccines such as Group B Streptococcus, Influenza and Pertussis in coastal Kenya.

## Conclusions

At least 77% of pregnant women from Kilifi HDSS, attending ANC would be reached for maternal RSV vaccination delivery through the ANC clinics at the currently optimum gestational age window. Concomitant administration of tetanus and RSV vaccine in the same period suggests 55% of women attending ANC would be available for uptake of both vaccines. Widening the vaccination window leads to a potential modest increase in vaccine coverage and its effect requires taking account of antibody kinetics data. Uncertainty in these estimates is due to 21% of women having no ANC card and reaching only 60% of our target population. Improving ANC attendance is a high priority for the success of a RSV maternal vaccine. These findings can be useful in guiding policy development towards implementation of a maternal RSV vaccine through the ANC platform in Kenya.

## Supplementary Information

**Additional file 1.** Pregnancy Assessment Form for KHDSS participants.

## Data Availability

The dataset and analysis scripts generated for this manuscript are available in Harvard Dataverse at 10.7910/DVN/TM8YHW. The data is stored under restricted access and available from the authors upon request through submission of a request form http://kemri-wellcome.org/aboutus/#ChildVerticalTab_15 for consideration by our Data Governance Committee (dgc@kemri-wellcome.org).

## Abbreviations

ANC: Antenatal care
DHS: Demographic Health Survey
RSV: Respiratory Syncytial Virus
KHDSS: Kilifi Health Demographic Surveillance System
KEMRI: Kenya Medical Research Institute
KWTRP: KEMRI Wellcome Trust Research Programme
KCH: Kilifi County Hospital
LMICs: Low- and Middle -Income Countries
SD: Standard deviation
WHO: World Health Organisation

## References

[1] PERCH (2019). Causes of severe pneumonia requiring hospital admission in children without HIV infection from Africa and Asia: the PERCH multi-country case-control study. Lancet.

[2] Berkley JA, Munywoki P, Ngama M, Kazungu S, Abwao J, Bett A, Lassauniere R, Kresfelder T, Cane PA, Venter M (2010). Viral etiology of severe pneumonia among Kenyan infants and children. Jama.

[3] Shi T, McAllister DA, O'Brien KL, Simoes EAF, Madhi SA, Gessner BD, Polack FP, Balsells E, Acacio S, Aguayo C (2017). Global, regional, and national disease burden estimates of acute lower respiratory infections due to respiratory syncytial virus in young children in 2015: a systematic review and modelling study. Lancet..

[4] Nokes DJ, Ngama MJ, Bett A, Abwao J, Munywoki P, English M, Scott JAG, Cane PA, Medley GF (2009). Incidence and severity of respiratory syncytial virus pneumonia in rural Kenyan children identified through hospital surveillance. Clin Infect Dis.

[5] Murphy BR, Alling DW, Snyder MH, Walsh EE, Prince GA, Chanock RM, Hemming VG, Rodriguez WJ, Kim HW, Graham BS (1986). Effect of age and preexisting antibody on serum antibody response of infants and children to the F and G glycoproteins during respiratory syncytial virus infection. J Clin Microbiol.

[6] Kim HW, Canchola JG, Brandt CD, Pyles G, Chanock RM, Jensen K, Parrott RH (1969). Respiratory syncytial virus disease in infants despite prior administration of antigenic inactivated vaccine. Am J Epidemiol.

[7] Karron RA, Buchholz UJ, Collins PL (2013). Live-attenuated respiratory syncytial virus vaccines. Curr Top Microbiol Immunol.

[8] Domachowske JB, Khan AA, Esser MT, Jensen K, Takas T, Villafana T, Dubovsky F, Griffin MP (2018). Safety, tolerability and pharmacokinetics of MEDI8897, an extended half-life single-dose respiratory syncytial virus Prefusion F-targeting monoclonal antibody administered as a single dose to healthy preterm infants. Pediatr Infect Dis J.

[9] Griffin MP, Yuan Y, Takas T, Domachowske JB, Madhi SA, Manzoni P, Simoes EAF, Esser MT, Khan AA, Dubovsky F (2020). Single-dose Nirsevimab for prevention of RSV in preterm infants. N Engl J Med.

[10] Glezen WP, Paredes A, Allison JE, Taber LH, Frank AL (1981). Risk of respiratory syncytial virus infection for infants from low-income families in relationship to age, sex, ethnic group, and maternal antibody level. J Pediatr.

[11] Roca A, Abacassamo F, Loscertales MP, Quinto L, Gomez-Olive X, Fenwick F, Saiz JC, Toms G, Alonso PL (2002). Prevalence of respiratory syncytial virus IgG antibodies in infants living in a rural area of Mozambique. J Med Virol.

[12] Englund J, Glezen WP, Piedra PA (1998). Maternal immunization against viral disease. Vaccine.

[13] Higgins D, Trujillo C, Keech C (2016). Advances in RSV vaccine research and development - a global agenda. Vaccine.

[14] PATH: **RSV vaccine and mAb snapshot. 2019**. https://www.pathorg/resources/rsv-vaccine-and-mab-snapshot (updated 28 August 2019) 2019.

[15] Novavax I: **N**ovavax Announces Topline Results from Phase 3 PrepareTM Trial of ResVax™ for Prevention of RSV Disease in Infants via Maternal Immunization**.** 2019. http://www.irnovavaxcom/news-releases/news-release-details/novavax-announces-topline-results-phase-3-preparetm-trial.

[16] Engmann C, Fleming JA, Khan S, Innis BL, Smith JM, Hombach J, Sobanjo-Ter Meulen A (2020). Closer and closer? Maternal immunization: current promise, future horizons. J Perinatol.

[17] Abu-Raya B, Giles ML, Kollmann TR, Sadarangani M (2019). The effect of timing of tetanus-diphtheria-Acellular pertussis vaccine Administration in Pregnancy on the avidity of pertussis antibodies. Front Immunol.

[18] Nyiro JU, Sande C, Mutunga M, Kiyuka PK, Munywoki PK, Scott JA, Nokes DJ (2015). Quantifying maternally derived respiratory syncytial virus specific neutralising antibodies in a birth cohort from coastal Kenya. Vaccine.

[19] Sande CJ, Mutunga MN, Okiro EA, Medley GF, Cane PA, Nokes DJ (2013). Kinetics of the neutralizing antibody response to respiratory syncytial virus infections in a birth cohort. J Med Virol.

[20] GOK (2014). Kenya Demographic Health Survey. Official report 2014, official DHS report.

[21] Nyiro JU, Munywoki P, Kamau E, Agoti C, Gichuki A, Etyang T, Otieno G, Nokes DJ (2018). Surveillance of respiratory viruses in the outpatient setting in rural coastal Kenya: baseline epidemiological observations. Wellcome Open Res.

[22] Scott JA, Bauni E, Moisi JC, Ojal J, Gatakaa H, Nyundo C, Molyneux CS, Kombe F, Tsofa B, Marsh K (2012). Profile: the Kilifi health and demographic surveillance system (KHDSS). Int J Epidemiol.

[23] Charan J, Biswas T (2013). How to calculate sample size for different study designs in medical research?. Indian J Psychol Med.

[24] MOH (2012). Kenya Ministry of Public Health and Sanitation and Ministry of Medical Services; National Guidelines for Quality Obstetrics and Perinatal Care.

[25] Hogan AB, Campbell PT, Blyth CC, Lim FJ, Fathima P, Davis S, Moore HC, Glass K (2017). Potential impact of a maternal vaccine for RSV: a mathematical modelling study. Vaccine.

[26] WHO (2016). WHO Guidelines Approved by the Guidelines Review Committee, in WHO Recommendations on Antenatal Care for a Positive Pregnancy Experience.

[27] Dako-Gyeke P, Aikins M, Aryeetey R, McCough L, Adongo PB (2013). The influence of socio-cultural interpretations of pregnancy threats on health-seeking behavior among pregnant women in urban Accra, Ghana. BMC pregnancy and childbirth.

[28] Abeje G, Azage M, Setegn T (2014). Factors associated with institutional delivery service utilization among mothers in Bahir Dar City administration, Amhara region: a community based cross sectional study. Reprod Health.

[29] Beyondzero (2014). NO WOMAN SHOULD DIE WHILE GIVING BIRTH.

[30] Government of Kenya, MOH. Guidelines for Conducting Adolescents Sexual and Reproductive Health Research in Kenya. Kenya: National AIDS and STI Control Programme (NASCOP) & Kenya Medical Research Institute (KEMRI); 2015. p. 13–5.

