# Supplementary material for: Implications of gestational age at antenatal care attendance on the successful implementation of a maternal respiratory syncytial virus (RSV) vaccine program in coastal Kenya
Source: BMC Public Health. 2020 Nov 16;20:1723. doi: 10.1186/s12889-020-09841-9 (PMC7670712; doi:10.1186/s12889-020-09841-9)
Supplement: Supplementary file 1 — Additional file 1. Pregnancy Assessment Form for KHDSS participants. [file 12889_2020_9841_MOESM1_ESM.docx]

## **Pregnancy Assessment Form for KHDSS participants**

**Study lay Title:** **A study to investigate factors that would influence the success of a maternal RSV vaccine program in Kenya.**

Date today (DD/MM/YYYY) __ __ /__ __/ 20__ _ Time: ___:___hrs

**Section I: Personal details (Extracted from KHDSS database)**

1. Study Person id: __ __ __ __ ___ __ DSS PID: __ __ __ __ __ __ Residence (Village): _____________ HM Name: __ __ __ __ __ __ ____ ____ __ __ __ __ Location:_ ___ __ __ __

**Section II: Antenatal Attendance (Fieldworker to fill)**

2. Mother attended ANC? [ ] Y/N

3. If No give reasons for not attending ANC: [ ] Reason1__ __ __ [ ] Reason2__ __ __ [ ] Reason3__ __ __ __ **End further data collection from the participant.**

4. If attended ANC is **YES**, is ANC booklet available? [ ] Y/N

5. If ANC booklet available is **NO** give reason: [ ] Lost or misplaced [ ] not issued

And proceed to fill in **Q7-Q9, Q11-Q15, Q17-Q21 and Q24-Q31** by asking the participant.

6. If ANC booklet available is **YES**, proceed to fill in the sections below using the booklet and ask the participant for information not in the booklet for **Q17-Q21** and **Q24-Q31**.

**Section III: Maternal Profile**

7. Facility ANC attended: __ __ __ __ ___ __

8. Participant Names: 1__________________ 2__________________ 3___________________

9. Age: __ __ __ __ ___ __ Gravida: __ __ __ __ ___ __ Parity: __ __ __ __ ___ __

10. LMP: __ __ __ __ ___ __ EDD: __ __ __ __ ___ __

11. Marital status: 1. Married [ ] 2. Single [ ] 3. Divorced/Separated [ ] 4. Widowed [ ]

12. Education level: 1. Some primary [ ] 2. Primary completed [ ] 3. Some Secondary [ ] 4. Secondary completed [ ] 5. Tertiary [ ] (Specify if College or University) 6. None [ ]

13. Occupation: 1. None [ ] 2. Employed (specify) [ ] 3. Farmer[ ] 4. Self employed /business woman [ ] 5. Casual labourer [ ] 6. Other [ ]

14. Ethnicity: __ __ __ ___ __ (Use KHDSS existing categories)

15. Religion: 1. Christian [ ] 2. Muslim [ ] 3. Other [ ]

**Section IV: Antenatal profile for pregnancy being assessed**

16**.** Antenatal profile table **(Extract information from Participant’s ANC booklet)**

| **ANC visit** | **ANC visit Date** | **Weight** | **Blood pressure** | **HB** | **Fundal Height** | **Adverse Events** | **Tetanus vaccine given** |
| --- | --- | --- | --- | --- | --- | --- | --- |
| **1** |  |  |  |  |  |  |  |
| **2** |  |  |  |  |  |  |  |
| **3** |  |  |  |  |  |  |  |
| **4** |  |  |  |  |  |  |  |
| **5** |  |  |  |  |  |  |  |

*For each ANC visit, have a breakdown of the following categories for adverse events and the fieldworker should tick those appearing in the booklet.

1. pre-eclampsia
2. eclampsia,
3. Malaria
4. gestational diabetes,
5. respiratory illness
6. reproductive tract infection
7. Other infections.

17. Do you know why you were given a tetanus vaccine during pregnancy? [ ] Y/N

18. If asked to take an injection during your pregnancy for a new vaccine to protect your child from pneumonia in the early months after birth, would you accept? [ ] Y/N

19. If answer is **NO**, give reasons: [ ] Reason1__ __ __ [ ] Reason2__ __ __ [ ] Reason3__ __ __ __

20. Would you need to consult anyone before taking a new vaccine injection during pregnancy? [ ] Y/N

21. If answer is YES, Who would you consult: 1. Spouse [ ] 2. Friends/relatives [ ] 3. Other (specify)

**Section V: Delivery details**

22. Date of delivery: __ __ __ __ _____ ___ Duration of pregnancy: __ __ __ __ _____ _(Weeks)

23. Mode of delivery: [ ] Normal Vaginal Delivery [ ] CS Baby’s weight at birth: __ __ __ __ __(Kgs)

24. Place of delivery: [ ] Health facility [ ] Home [ ] Other (specify)

25. If delivery occurred at home, give reasons: 1. Doctors strike [ ] 2. Distance to health facility [ ] 3. Cost [ ] 4. Quality of care / services [ ] 5. Other (specify) [ ]

26. If delivery occurred in health facility specify name of facility: __ __ __ __ __

29. Pregnancy outcome: [ ] Normal [ ] low birthweight [ ] premature [ ] Still birth

30. Adverse events at birth

*Have a breakdown of the following categories for adverse events at birth.

1. pre-eclampsia
2. eclampsia,
3. Malaria
4. Maternal Hemorrhage
5. other condition (specify).

31. What reasons made you attend for ANC services during pregnancy and choice of the place to deliver your baby? 1. Previous pregnancy history [ ] 2. Free services [ ] 3. Pregnancy complications [ ] 4. Friends/Relatives [ ] 5. Wellbeing of mother and child [ ] 6. Other [ ] (specify) __ __ __ __ __

32. Who made the decision for you to attend for ANC services during pregnancy or choice of the place to deliver your baby? 1. Self [ ] 2. Spouse [ ] 3. Both husband and wife [ ] 4. Community health worker [ ] 5. Relatives [ ] 6. Other [ ] (specify) __ __ __ __ __

**Initials of the Field worker collecting the data [ ] [ ]**
